# Probiotic Formula Ameliorates Renal Dysfunction Indicators, Glycemic Levels, and Blood Pressure in a Diabetic Nephropathy Mouse Model

**DOI:** 10.3390/nu15122803

**Published:** 2023-06-19

**Authors:** Yi-Wei Kuo, Yen-Yu Huang, Shin-Yu Tsai, Jiu-Yao Wang, Jia-Hung Lin, Zun-Jie Syu, Hui-Shan Wang, Yu-Chieh Hsu, Jui-Fen Chen, Ko-Chiang Hsia, Hsieh-Hsun Ho

**Affiliations:** 1 Department of Research and Design, Glac Biotech Co., Ltd., Tainan 744, Taiwan; vic.kuo@glac.com.tw (Y.-W.K.); jim.huang@glac.com.tw (Y.-Y.H.); shin-yu.tsai@glac.com.tw (S.-Y.T.); jiahung.lin@glac.com.tw (J.-H.L.); zunjie.syu@glac.com.tw (Z.-J.S.); jennifer.wang@glac.com.tw (H.-S.W.); yuchieh.hsu@glac.com.tw (Y.-C.H.); juifen.chen@glac.com.tw (J.-F.C.); shawn.hsia@glac.com.tw (K.-C.H.); 2 Center of Allergy, Immunology, and Microbiome (AIM), China Medical University Children’s Hospital, Taichung 404, Taiwan; wangjy@mail.cmu.edu.tw; 3 Allergy and Clinical Immunology Research (ACIR) Center, National Cheng Kung University, Tainan 701, Taiwan; 4 Department of Allergy and Immunology, China Medical University Children’s Hospital, Taichung 404, Taiwan

**Keywords:** probiotics, diabetic nephropathy, anti-inflammation, antioxidation, short-chain fatty acids (SCFAs)

## Abstract

One-third of patients with end-stage chronic kidney disease (CKD) experience diabetic nephropathy (DN), which worsens the progression of renal dysfunction. However, preventive measures for DN are lacking. *Lactobacillus acidophilus* TYCA06, *Bifidobacterium longum* subsp. *infantis* BLI-02, and *Bifidobacterium bifidum* VDD088 probiotic strains have been demonstrated to delay CKD progression. This study evaluated their biological functions to stabilize blood-glucose fluctuations and delay the deterioration of renal function. The db/db mice were used to establish a DN animal model. This was supplemented with 5.125 × 10^9^ CFU/kg/day (high dose) or 1.025 × 10^9^ CFU/kg/day (low dose) mixed with probiotics containing TYCA06, BLI-02, and VDD088 for 8 weeks. Blood urea nitrogen (BUN), serum creatinine, blood glucose, and urine protein were analyzed. Possible mechanisms underlying the alleviation of DN symptoms by probiotic strains were evaluated through in vitro tests. Animal experiments revealed that BUN, serum creatinine, and blood glucose upon probiotic administration were significantly lower than in the control group. The rate of change of urine protein decreased significantly, and blood pressure, glucose tolerance, and renal fibrosis were improved. In vitro testing indicated that TYCA06 and BLI-02 significantly increased acetic acid concentration. TYCA06, BLI-02, and VDD088 were associated with better antioxidation, anti-inflammation, and glucose consumption activities relative to the control. A combination of the probiotics TYCA06, BLI-02, and VDD088 attenuated renal function deterioration and improved blood-glucose fluctuation in a diabetes-induced CKD mouse model.

## 1. Introduction

Chronic kidney disease (CKD) refers to the long-term irreversible degradation of kidney function, and it affects approximately 10% of the adult population [1]. It typically occurs as a complication of other metabolic diseases. Diabetic nephropathy (DN) is a major cause of end-stage renal disease, and one-third of patients with end-stage CKD have comorbid type-2 diabetes [2,3]. Chronic inflammation and oxidative stress play significant roles in the development of diabetic nephropathy (DN) and its complications. Prolonged high blood glucose levels in individuals with diabetes lead to the excessive production of free radicals, which ultimately progress into diabetic kidney disease (DKD) [4]. 

Studies have indicated that a reducing oxidative stress damage within the body thereby reduces the risk of renal failure and diabetic kidney disease progression. Furthermore, supplementation with probiotics can alleviate inflammation and oxidative stress symptoms in individuals with DN [5,6]. Patients with end-stage CKD with severely damaged kidneys require dialysis and kidney transplantation [7]. Additionally, patients with CKD typically exhibit altered intestinal microbiota. In the guts of patients with CKD, reductions in several *Bifidobacterium* and *Lactobacillus* species have been observed and increases in pathogenic bacterial species have been reported; these changes were associated with elevated levels of inflammatory cytokines, endotoxins, and other uremic toxins [8]. Therefore, probiotic intervention may be a potential therapy for CKD management [9].

Probiotics are defined as “live microorganisms which when administered in adequate amounts confer a health benefit on the host” by the International Scientific Association for Probiotics and Prebiotics [10]. Numerous studies have reported that probiotics benefit human health by improving intestinal barrier function, limiting the growth of pathogens, and regulating immune function [11]. Additionally, probiotics may slow the progression of CKD and partially restore renal function (determined through estimated glomerular filtration rate) [12,13]. However, it remains unclear whether probiotics ameliorate the symptoms of end-stage CKD with comorbid type-2 diabetes or DN.

Numerous studies have indicated that probiotic-secreted short-chain fatty acids (SCFAs) are involved in metabolic regulation, including the regulation of glycemic levels in people with type-2 diabetes [14], reduction of renal inflammation and damage in CKD mice [15], mediation of host inflammatory effects [16], and reduction of oxidative stress in aged animals [17]. Therefore, we hypothesized that probiotics would alleviate the symptoms of DN through the secretion of SCFAs.

Based on the basis of our previous research, we selected three probiotic strains through indole assay in vitro, and the results showed that *Lactobacillus acidophilus* TYCA06, *Bifidobacterium longum* subsp. *infantis* BLI-02, and *B. bifidum* VDD088 exhibited almost no indole production in the culture media. We hypothesized that combining these three probiotics could potentially attenuate renal function deterioration in both CKD mice and human patients. In the study, we observed several positive outcomes. Firstly, there was a reduction in serum levels of endotoxin, TNF-α, IL-6, and IL-18, indicating a decrease in inflammation. Additionally, we noted improvements in stool form, borborygmus, and flatulence. Furthermore, there was an increased abundance of *B. bifidum* and *B. breve* in the human stool microbiota [13]. Previous experiments primarily focused on investigating the impact of probiotics on human chronic kidney disease and mouse animal models induced by adenine [18]. Therefore, this study aimed to simulate chronic kidney disease using the db/db mouse model and explore whether these three renal-protective strains (TYCA06, BLI-02, and VDD088) could delay the progression of CKD associated with type-2 diabetes or DN [19]. Moreover, we aimed to evaluate the potential metabolic mechanisms of probiotic supplementation, including antioxidative activity, anti-inflammation, glucose consumption ability, and SCFA secretion.

## 2. Materials and Methods

### 2.1. Probiotic Strains and Cultivation

Active and dry *L. acidophilus* TYCA06, *B. longum* subsp. *infantis* BLI-02, and *B. bifidum* VDD088 were obtained from Glac Biotech (Tainan, Taiwan). *L. acidophilus* TYCA06 was isolated from healthy human intestines and deposited at the China General Microbiological Culture Collection Center (CGMCC 15210, Beijing, China) and Bioresource Collection and Research Center (BCRC 910813, Hsinchu, Taiwan). *B. longum* subsp. *infantis* BLI-02 was isolated from the breasts of healthy human individuals and deposited at the China General Microbiological Culture Collection Center (CGMCC 15212, Beijing, China) and Bioresource Collection and Research Center (BCRC 910812, Hsinchu, Taiwan). *B. bifidum* VDD088 was isolated from healthy infant intestines and deposited at the China General Microbiological Culture Collection Center (CGMCC 15211, Beijing, China) and Bioresource Collection and Research Center (BCRC 910814, Hsinchu, Taiwan).

Three viable strains were cultured with de Man, Rogosa, and Sharpe (MRS) broth containing 0.05% cysteine under anaerobic conditions at 37 °C for 20 h. A CFU assay was used to measure probiotic viability. The probiotic strains were dried through lyophilization. The concentration of live strains was 10^11^ CFU/g, and these were mixed in a ratio of 1:1:1. Subsequently, the dry powder of mixed strains (TYCA06, BLI-02, and VDD088) was made into capsules with concentrations of 2.5 × 10^9^ CFU in ratios of 1:1:1.

### 2.2. DN Animal Model and Probiotic Intervention

The C57BL/6 mice (8 weeks of age, 6 males per group), presented as db/m mice, were selected as the naive group. BKS Cg-Dock 7m +/+ Leprdb/ JNarl (db/db) mice (8 weeks of age, 12 males per group), which were induced with high blood glucose for chronic kidney failure, were used as the probiotic intervention group. The animal study was conducted in National Cheng Kung University Hospital (Tainan, Taiwan). All the animals were housed and treated in compliance with the National Institutes of Health Guide for the Care and Use of Laboratory Animals. The Animal Ethics Committee and the Institutional Animal Care and Use Committee of the College of Medicine, National Cheng Kung University approved the protocols of these animal experiments (IACUC No. 107024).

The probiotic intervention groups were fed a probiotic combination daily (high dose: 5.125 × 10^9^ CFU/kg/day; low dose: 1.025 × 10^9^ CFU/kg/day) through an oral gavage for 2 months (8 weeks of age, 12 males per group; db/db mice). The BKS. Cg-Dock 7m +/+ Leprdb/JNarl mice in the control group (8 weeks of age, 12 males; db/db mice) were fed sterilized food without probiotic supplementation. All animals were sacrificed, and their kidney tissue was collected on day 57. Researchers collected the blood samples of the mice on day 1 of the experiment and 1 day before the sacrifice for analysis. 

### 2.3. Biochemical Analysis

The body weights of all mice were measured once a week during the experiment. The tails of all the mice were fixed for measuring blood pressure at week 8 using a noninvasive continuous blood pressure monitor (Visitech BP-2000 System; Visitech Systems, Apex, NC, USA). To enable mice to adapt to the restricted activity, all mice were habituated and experienced simulated blood pressure for 3 days before the end of the blood pressure test. An oral glucose tolerance test (OGTT) was performed on all mice before the probiotic intervention, and the fasting blood glucose (glucose AC), blood urine nitrogen (BUN), blood creatinine, and urine protein of all mice were measured every 2 weeks before and after probiotic supplementation. OGTTs were conducted and postprandial blood glucose were measured in all mice at week 8.

All mice fasted overnight before the measurement of glucose AC or OGTT results, and blood samples were collected from their tail veins. The routine OGTT proceeded as follows: all mice were administered oral glucose (2 g/kg), and tail vein blood was collected at intervals of 30, 60, 90, and 120 min. All collected blood or urine samples were placed on test strips and analyzed for blood glucose, BUN, blood creatinine, and urine protein concentrations with a veterinary chemistry analyzer (FUJI DRI-CHEM 4000i; Fuji film Corporation, Tokyo, Japan).

### 2.4. Tissue Sectioning and Staining

The kidney tissues of the mice were fixed in 10% neutral formalin for 24 h. A graded series of ethanol was used to dehydrate the fixed organs. The prepared kidney tissues were embedded in paraffin blocks. Hematoxylin–eosin staining was performed for observations of glomeruli appearance in kidney sections (magnification, 100×; bar, 20 µm). Masson’s trichrome staining was used to detect fibrosis in the kidneys (renal cortex; magnification, 40×; bar, 200 µm). The experimental protocol was identical to that in a previous study [13].

### 2.5. In Vitro Analysis of Oxidative Stress

A stable nitrogen-centered radical, DPPH (2,2-diphenyl-1-picryl-hydrazyl-hydrate; Sigma-Aldrich, St. Louis, MO, USA), was used to detect antioxidative activity. A 0.2 mM DPPH in methanol solution was added with 2 × 10^9^ CFU probiotic solution, 10 μg/mL of vitamin C (positive control), or medium (blank group), and they were mixed in a ratio of 1:1. The test solution was incubated for 30 min in the dark at room temperature and then centrifuged at 12,000 rpm for 2 min at 4 °C. The tested solutions were measured at an absorbance of 517 nm with a μQuant microplate spectrophotometer (BioTek, Santa Clara, CA, USA). The calculation formula is as follows:

The DPPH scavenging ratio = (OD_blank − OD_sample)/OD_blank × 100%

where OD_Blank is the absorbance value of the control medium and OD_sample is the absorbance value of the tested samples.

### 2.6. In Vitro Anti-Inflammatory Cytokine IL-10 Assays

The low-density fraction (approximately 42.5–50% interface) of healthy peripheral blood mononuclear cells (PBMCs; Blood Center, Taiwan Blood Services Foundation) were isolated using the Ficoll–Hypaque gradient (Pharmacia, Uppsala, Sweden) at a 3000-rpm centrifuge spin rate for 10 min. The supernatant was discarded and 100 μL of 4 × 10^5^ cells/100 μL/well PBMCs were then seeded into 96-well plates (No. 167008, MicroWell 96-Well; Thermo-Fisher, Waltham, MA, USA). Subsequently, 20 μL of the probiotics (4 × 10^6^ CFU) were added at ratio of 1:10 (postbiotics: PBMCs) into PBMC cell-culture plates at 37 °C for 48 h (5% CO_2_). In addition, 20 μL of cell-culture medium was co-cultured with PBMCs to serve as the medium for the control group, and 20 μL of Phytohemagglutinin (PHA; 0.2 μg/mL; Thermo-Fisher, Waltham, MA, USA) co-cultured with PBMCs served as the positive control group. Finally, the co-culturing plate was centrifuged at 3000 rpm for 10 min at 4 °C. Anti-inflammatory cytokines IL-10 were measured through ELISA assay (eBioscience) using a μQuant microplate spectrophotometer (BioTek, Santa Clara, CA, USA) at an absorbance value of 450 nm. The experimental protocol was identical to that in a previous study [20].

### 2.7. In Vitro Glucose Consumption Assay

Probiotics (1 × 10^8^ CFU/0.1 mL) were mixed with a 5-mL MRS broth containing 2% glucose (Thermo-Fisher, Waltham, MA, USA) and incubated at 37 °C for 8 h; 0.05% of cysteine (Thermo-Fisher, Waltham, MA, USA) was added to the medium when culturing the *Bifidobacterium* species. The probiotic strain *L. casei* gL-10 was obtained from Glac Biotech (Tainan, Taiwan) and was used as a positive control (potential strains of glucose consumption discovered by our previous lab research). The supernatant was collected through centrifugation at 12,000 rpm for 5 min. A 3,5-dinitrosalicylic acid (DNS) assay (Thermo-Fisher, Waltham, MA, USA) was used to evaluate the reduction of sugars in the supernatant. The absorbance was measured at a wavelength of 540 nm. The standard curve of the glucose standard solution was used to measure the concentration of glucose in the probiotic supernatant. The experimental protocol was performed following that reported in previous research [21]. The formula for calculating the percentage of glucose consumption is as follows:

The glucose consumption ratio (%) = (glucose content before probiotic culture-glucose content after probiotic culture)/glucose content before probiotic culture × 100%.

### 2.8. Measurement of Short-Chain Fatty Acids

In total, 50 μL of 50% sulfuric acid, 10 μL internal standard, and 200 μL ether were added to 150 μL of the individual probiotic supernatant. The mixture was shaken for 15 min and then centrifuged at 9000 rpm for 10 min at 4 °C. The ether layer was detected using GS/MS (Agilent 7890 gas chromatography mass spectrometer equipped with Agilent HP-FFAP capillary column, 30 m × 250 μm × 0.25 μm; Santa Clara, CA, USA) after adding anhydrous sodium sulfate (Na_2_SO_4_) to the ether layer for dehydration. GS/MS analysis was used to detect levels of SCFAs (µmol/mL) including acetic acid, propanoic acid, isobutyric acid, butyric acid, and pentanoic acid. The experimental protocols followed those of a previous study [17]. 

### 2.9. Statistical Analysis

The differences between continuous variables were analyzed using the Mann–Whitney *U* test. Statistical significance was indicated by *p* < 0.05. Continuous variables were expressed in terms of means ± standard deviation. All analyses were conducted using SPSS (IBM, Armonk, NY, USA).

## 3. Results

### 3.1. The db/db Mice Successfully Presented the Clinical Symptoms of Diabetic Nephropathy

**Figure 1:**
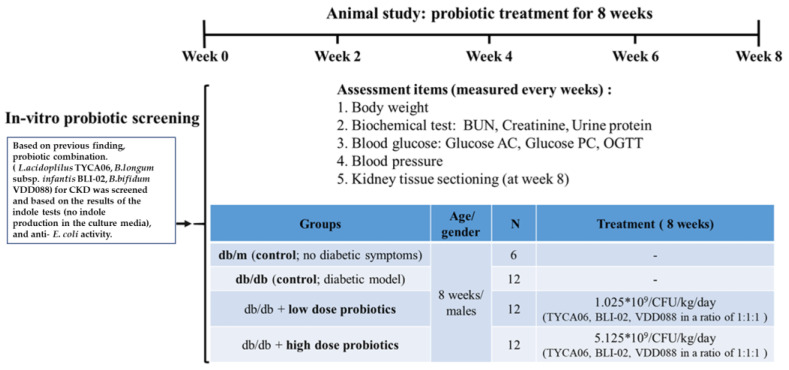
The experimental design flowchart for the probiotic-alleviated DN animal model [10].

The experimental design flowchart for the probiotic-alleviated DN animal model is presented in Figure 1. The db/db mice were used to mimic DN symptoms. In comparison with the normal mice group (db/m group), the db/db mice had significantly higher body weight, poorer blood glucose control, higher blood pressure, worse renal function, kidney shrinkage, the formation of collagen fibrosis in the renal cortex, and the formation of vacuolation in the glomerulus. 

### 3.2. The Probiotic Formula Improved the Blood Glucose Levels among Diabetic Nephropathy-Induced Mice

**Figure 2:**
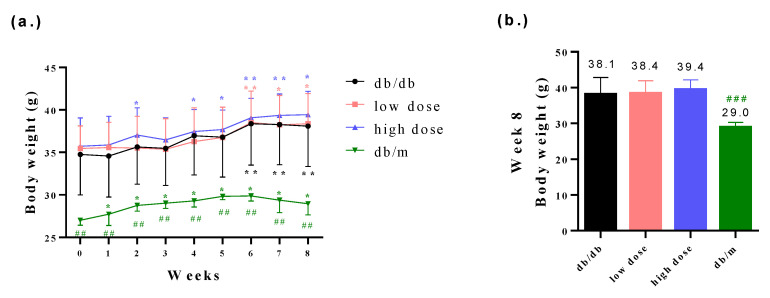
(**a**) The body weight changes among all groups from week 0 to week 8 and (**b**) the measurement of body weights among all groups at week 8. * *p* < 0.05, ** *p* < 0.01 indicate statistically significant differences in comparison with week 0. ## *p* < 0.01, ### *p* < 0.001 indicate statistically significant differences in comparison with the db/db group.

**Figure 3:**
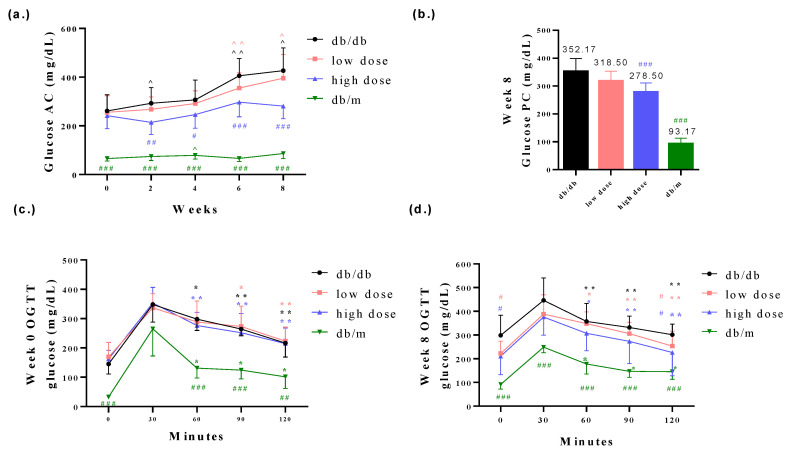
The probiotic formula mitigated (**a**) glucose AC level, (**b**) glucose PC level and (**c**,**d**) OGTT in db/db mice. ^ *p* < 0.05, ^^ *p* < 0.01 indicate statistically significant differences in comparison with week 0. * *p* < 0.05, ** *p* < 0.01 indicate statistically significant differences in comparison with 30 min. # *p* < 0.05, ## *p* < 0.01, ### *p* < 0.001 indicate statistically significant differences in comparison with the db/db group.

The body weights of db/db mice were found to be higher compared to those of db/m mice. However, the probiotic intervention among the db/db mice did not affect the body weights of the db/db mice (Figure 2a,b). Furthermore, it is worth noting that db/db mice generally exhibit poorer blood glucose control compared to db/m mice. The probiotic intervention led to improved glycemic control in the db/db mice (Figure 3a–d). Fasting plasma glucose (AC) refers to the measurement of glucose levels in the blood after an overnight fast. It is typically measured in the morning before breakfast, after at least 8 h of fasting. AC levels provide an indication of the baseline blood glucose concentration and are commonly used to diagnose and monitor diabetes. In comparison with the db/db-mouse control group, the high-dose probiotic treatment group exhibited significantly reduced glucose AC to 214.4 mg/dL (## *p* < 0.01), 246.2 mg/dL (# *p* < 0.05), 297.5 mg/dL (### *p* < 0.001), and 281.3 mg/dL (### *p* < 0.001) at weeks 2, 4, 6, and 8, respectively (Figure 3a). Postprandial plasma glucose (PC) refers to the measurement of glucose levels in the blood after a meal. It is taken a certain period of time after eating, typically two hours. PC levels help assess the body’s ability to regulate glucose levels after consuming food. The high-dose probiotic treatment group had significantly decreased glucose PC to 278.5 mg/dL (### *p* < 0.001) at week 8 (Figure 3b). The OGTT is used to assess glucose tolerance and observe changes in blood glucose levels in mice over time. It also provides insights into alterations in insulin secretion relative to time, indicating insulin resistance. Compared to the db/db group, the high-dose probiotic group did not show any improvement in OGTT levels at week 0 (Figure 3c). However, after 8 weeks of supplementation, there was a significant decrease in OGTT levels in the probiotic group (to 210.6 mg/dL at 0 min and to 216.5 mg/dL at 120 min; # *p* < 0.05) (Figure 3d). This indicates that probiotic supplementation effectively lowers blood glucose levels, demonstrating thar these three probiotics have potential benefits in blood glucose control.

### 3.3. Probiotic Formula Reduced the Blood Pressure among Diabetic Nephropathy Induced Mice

**Figure 4:**
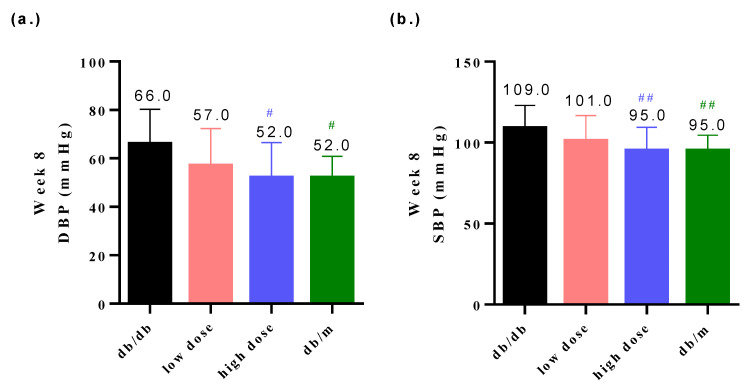
The probiotic formula attenuated (**a**) the diastolic blood pressure (DBP) and (**b**) systolic blood pressure (SBP) in db/db mice at week 8. # *p* < 0.05, ## *p* < 0.01 indicate statistically significant differences in comparison with the db/db group.

High blood pressure is a clinical indicator of DN [22]. The groups that were administered low or high doses of the probiotic intervention exhibited decreased blood pressure levels relative to the db/db group (Figure 4a,b). The low-dose probiotic supplementation significantly reduced the diastolic blood pressure (DBP) level to 57.0 mmHg (*# p* < 0.05) at week 8 (Figure 4a) and the high-dose probiotic reduced the DBP level to 52.0 mmHg (*# p* < 0.05) at week 8 (Figure 4a); the low-dose probiotic supplementation significantly reduced the systolic blood pressure (SBP) level to 101.0 mmHg (#*# p* < 0.01) at week 8 (Figure 4b) and the high-dose probiotic reduced the SBP level to 95.0 mmHg (#*# p* < 0.01) at week 8 (Figure 4b).

### 3.4. Probiotic Formula Decreased the Renal Dysfunction Levels

**Figure 5:**
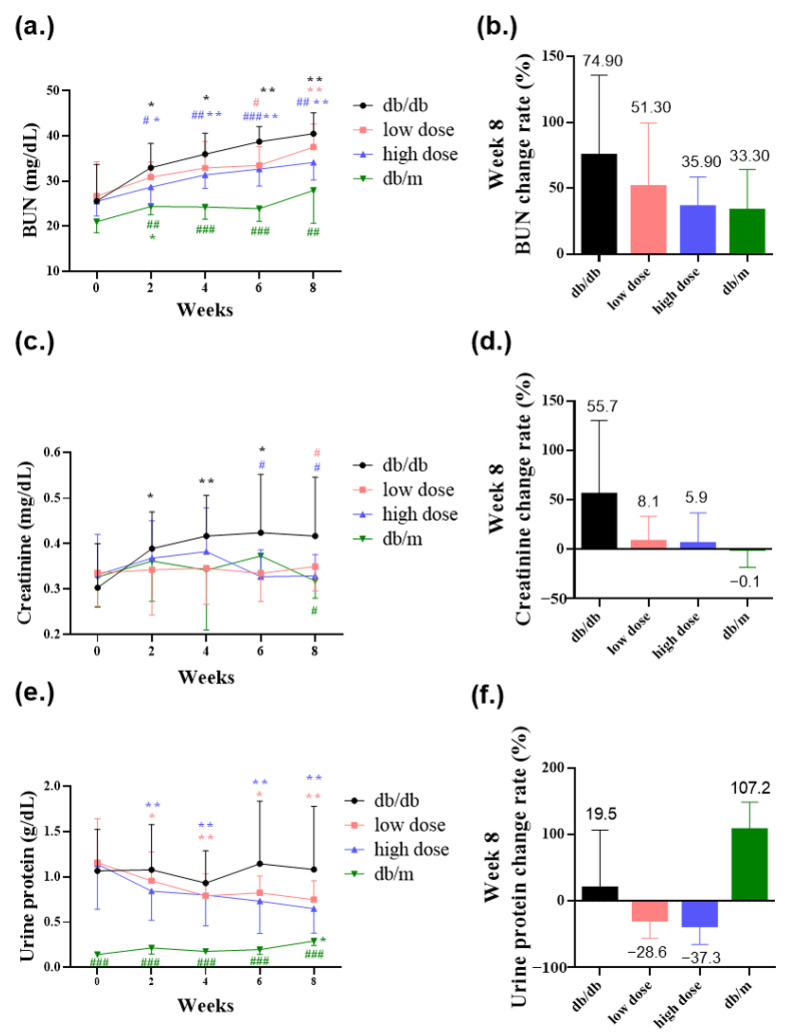
The probiotic formula ameliorated diabetic nephropathy revealed in (**a**) blood urea nitrogen (BUN) level (mg/dL), (**b**) blood urea nitrogen (BUN) rate of change (%) at week 8, (**c**) creatinine level (mg/dL), (**d**) creatinine rate of change (%) at week 8, (**e**) urine protein level (g/dL), and (**f**) urine protein rate of change (%) at week 8. * *p* < 0.05, ** *p* < 0.01, indicate statistically significant differences in comparison with week 0. # *p* < 0.05, ## *p* < 0.01, ### *p* < 0.001 indicate statistically significant differences in comparison with the db/db group.

BUN, creatinine, and urine protein are key clinical indicators of renal function. Elevated levels of BUN, creatinine, and urine protein are associated with deteriorating renal function, indicating impaired filtration of nitrogenous waste in the kidneys [23]. In comparison to the db/m mice group, db/db mice exhibited poorer renal function. However, when comparing to the db/db mice group, the group that received the high-dose probiotic had significantly reduced BUN levels to 28.7 mg/dL (# *p* < 0.05), 31.4 mg/dL (## *p* < 0.01), 32.7 mg/dL (### *p* < 0.001), and 34.1 mg/dL (## *p* < 0.01) at weeks 2, 4, 6, and 8, respectively (Figure 5a,b). The high-dose probiotic significantly decreased the rate of change of BUN to 35.9% at week 8 (Figure 5b). The low-dose probiotic significantly decreased the BUN level to 33.5 mg/dL (## *p* < 0.01) at week 6 (Figure 5a).

The high-dose probiotic significantly decreased creatinine levels to 0.33 mg/dL (# *p* < 0.05) at week 6 and 0.33 mg/dL (# *p* < 0.05) at week 8 (Figure 5c). The low-dose probiotic significantly decreased creatinine levels to 0.35 mg/dL (# *p* < 0.05) at week 8 (Figure 5c). The low-dose and high-dose probiotics decreased the rate of change of creatinine at week 8 to 8.1% and 5.9%, respectively (Figure 5d). Additionally, the high-dose probiotic decreased urine protein levels and the rate of change of urine protein to 0.65 g/dL and −37.3%, respectively, at week 8 (Figure 5e,f). 

**Figure 6:**
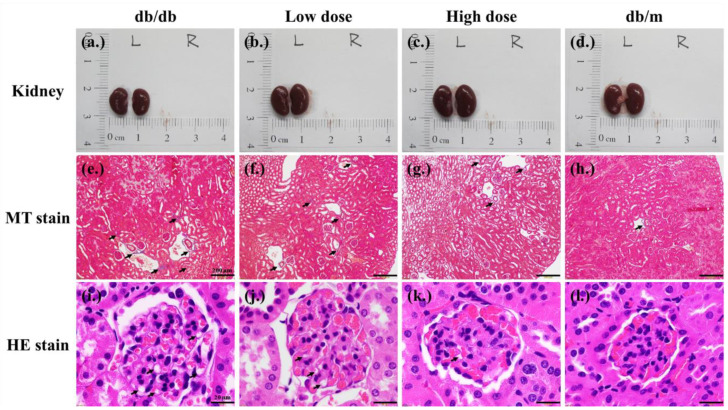
Effects of 8 weeks of probiotic supplementation on kidney appearance (**a**–**d**). Compared to db/m mice, the kidneys of db/db mice are smaller. Both low-dose and high-dose probiotic supplementations help maintain the kidney sizes of db/db mice (**e**–**h**). The Masson’s trichrome staining revealed that probiotic formula attenuated the collagen fibrosis of kidneys (renal cortex) in db/db mice. Each black arrow indicates a collagen fibrosis that was stained blue (magnification, 40×; bar, 200 µm). (**i**–**l**) A Hematoxylin–Eosin (HE) stain revealed that probiotic formula attenuated the formation of the vacuolation of kidneys (glomerulus) in db/db mice. The black arrows indicate the vacuolation sites (magnification, 100×; bar, 20 µm).

In kidney histological sections, it was observed that the kidneys of db/db mice, due to renal damage, were slightly smaller than those of db/m mice and had a darker red color. Additionally, there was evidence of collagen fibrosis in the renal cortex and vacuolation in the glomerulus. The low and high doses of probiotic supplementations decreased the collagen fibrosis of the renal cortex (Figure 6f,g) and the formation of glomeruli vacuolation (Figure 6j,k).

### 3.5. Potential Mechanisms of Probiotic Effect on Diabetic Nephropathy

**Figure 7:**
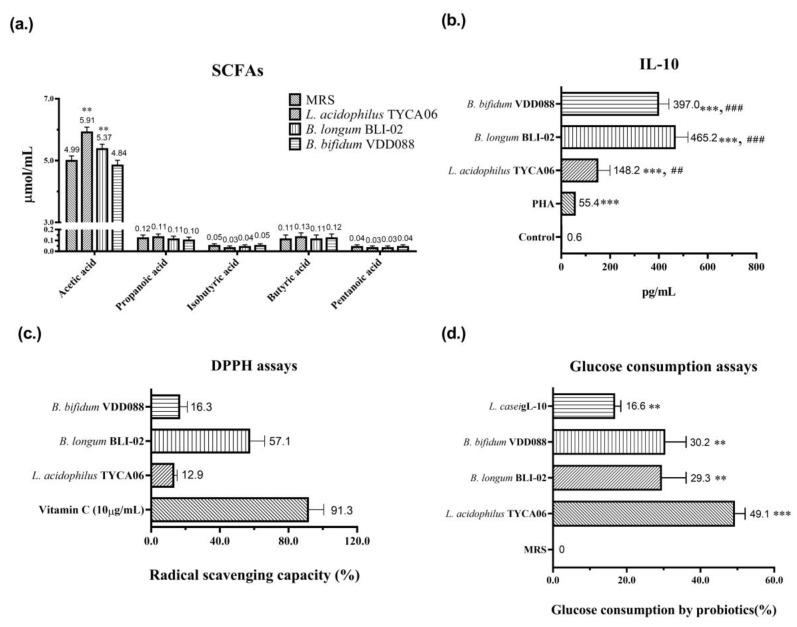
In vitro analysis of (**a**) short-chain fatty acids (SCFAs), where ** *p* < 0.01 indicate statistically significant differences in comparison with MRS, (**b**) anti-inflammatory cytokine—IL-10, where *** *p* < 0.001 indicates statistically significant differences in comparison with control and ## *p* < 0.01 and ### *p* < 0.001 indicate statistically significant differences in comparison with PHA, (**c**) antioxidative activity (DPPH assay), and (**d**) glucose consumption ability suggests the possible mechanisms through which probiotics meditated the alleviation of diabetic nephropathy. The PHA, vitamin C, and *L. casei* gL-10 were used as the positive controls in the IL-10 assay, DPPH assay, and glucose consumption assay, respectively.

Subsequently, several in vitro tests were performed to investigate the possible mechanisms underlying the modulation of DN with probiotic formula. Acetate salt is one of the main components of short-chain fatty acids (SCFAs). The literature indicates that acetate, butyrate, and isovalerate are negatively correlated with DN [24]. Acetate can impact brain function and metabolism in individuals with type-2 diabetes, providing benefits for T2D [25]. Two strains of the probiotic formula, *L. acidophilus* TYCA06 and *B. longum* BLI-02, significantly elevated acetic acid levels to 5.91 µmol/mL (** *p* < 0.01) and 5.37 µmol/mL (** *p* < 0.01), respectively (Figure 7a). Additionally, *B. bifidum* VDD088, *B. longum* BLI-02, and *L. acidophilus* TYCA06 significantly increased anti-inflammatory cytokine IL-10 levels to 397.0 pg/mL (### *p* < 0.001), 465.2 pg/mL (### *p* < 0.001), and 148.2 pg/mL (## *p* < 0.01), respectively (Figure 7b). Furthermore, *B. longum* BLI-02 induced a 57.1% increase in antioxidative activity in the DPPH assay (Figure 7c). Reducing chronic inflammation and oxidative stress can potentially benefit the treatment of DN [26,27,28]. *L. acidophilus* TYCA06 induced a 49.1% increase in glucose digestion ability, as indicated in glucose consumption assays (Figure 7d).

## 4. Discussion

Previous research has reported that the combination of probiotics TYCA06, BLI-02, and VDD088 can effectively attenuate renal function deterioration [13], but it has remained undetermined whether they could alleviate the symptoms of DN. High blood glucose levels attributable to DN damage the blood vessels and renal cells of patients with CKD and leads to high blood pressure, renal dysfunction, and the progression of end-stage renal disease [2,3]. Therapeutic options for preventing the progression of DN remain insufficient. Jiang et al. reported that the glycemic control of patients with DN was ameliorated through probiotic supplementation [29]. Another study indicated that aerobic exercise training could effectively regulate oxidative stress and inflammatory cytokines, leading to improved kidney function in individuals with diabetic nephropathy [27]. However, neither of these studies provided further discussion or exploration of the potential mechanisms by which probiotics may exert their beneficial effects in DN.

Prior to the probiotic intervention, the animals underwent an adaptation period, and measures were taken to minimize stress and potential data inaccuracies caused by the mice’s responses to handling procedures. In this animal study, the combination of probiotics TYCA06, BLI-02, and VDD088 effectively stabilized glycemic levels including those of glucose AC, glucose PC, and OGTTs, especially in the high-dose probiotic supplementation group (Figure 3). The in vitro glucose consumption activity of TYCA06, BLI-02, and VDD088 may be one of the possible reasons underlying the regulation of glycemic levels in the DN mouse model (Figure 7d). However, whether TYCA06, BLI-02, and VDD088 ameliorate hyperglycemia through modulating insulin levels and protecting islet cells in db/db mice should be investigated [30]; similarly, whether TYCA06, BLI-02, and VDD088 reduce blood pressure by regulating plasma vasopressin (also known as the antidiuretic hormones) or the renin–angiotensin system should be verified in future research (Figure 4a,b) [31].

Previous studies have presumed that TYCA06, BLI-02, and VDD088 delayed CKD progression in adenine-induced CKD mice and patients with CKD by inhibiting the growth of intestinal-indole-producing pathogens [13]. Similarly, three probiotic strains attenuated renal function deterioration in DN mice in the present study (Figure 5 and Figure 6). The blood vessels in the kidneys and the nephrons were easily damaged by the accumulated blood-glucose concentrations in DN mice, which can damage the normal function of the kidneys. The three combined strains may ameliorate kidney damage in DN mice by regulating glycemic levels (Figure 3a–d and Figure 6). In this study, the probiotic strains TYCA06, BLI-02, and VDD088 demonstrated antioxidative activity in vitro, which could potentially contribute to the delayed progression of DN (Figure 7c). Additionally, Nagase et al. suggested that intestinal functional microenvironments are involved in DN progression, including reactive oxygen species biosynthesis and levels of SCFAs [32]. However, it should be noted that the efficacy of these probiotic strains in mitigating DN progression may vary among individuals due to various factors such as personal characteristics, dietary habits, lifestyles, and genetic factors. Therefore, additional clinical research is needed to determine effective intervention measures for DN.

The literature has highlighted the beneficial effects of probiotic-secreting SCFAs for CKD treatment [33]. SCFAs produced by gut microbiota can reduce lipopolysaccharide and activate a proinflammatory cascade [34]. Furthermore, the study suggests an association between the IL-1β/Caspase-1 cytokine cascade and the pathogenesis of early-stage nephropathy in relation to obesity and diabetes [35]. It may be valuable to consider the role of inflammatory cytokines in triggering the early stages of nephropathy, in addition to their involvement in the advanced stages. The mechanisms of SCFA-ameliorated DN progression are possibly the induction of glucagon-like peptide 1 [36], regulation of G protein-coupled receptors on endocrine cells [37], protection of renal cells from oxidative stress [38], and inhibition of histone deacetylases [39]. Present research indicates that TYCA06 and BLI-02 significantly elevate acetic acid levels, while TYCA06, BLI-02, and VDD088 increase anti-inflammatory cytokine levels in vitro (Figure 7a,b). Accordingly, Lactobacillus strains delayed the progression of CKD in the mice by elevating the SCFA levels, resulting in a reduction of renal inflammation and damage. However, these results for SCFA changes were not observed in a clinical trial [15]. Additionally, some clinical studies reported that probiotic supplementation ameliorated CKD progression, but these studies did not explore the long-term effects of probiotic administration [13,40].

Moreover, it has been reported that high glucose exposure with the overproduction of reactive oxygen species leads to renal podocyte apoptosis in DN [41] and the pathogenesis of nephropathy [42]. Some animal experiments have reported that antioxidants including ascorbic acid, resveratrol, and ubiquinone present therapeutic benefits for DN [43]. In this study, probiotic strains of TYCA06, BLI-02, and VDD088 exhibited antioxidative activity to some extent in vitro, which may partially contribute to the delay in the progression of DN (Figure 7c).

Despite the therapeutic potential of these three combined probiotic strains for DN, this study had several limitations. The SCFAs, antioxidative activity, and anti-inflammatory cytokines in the serum should have been tested further. Future studies should determine whether the intestinal microbiota of DN mice are altered by probiotic intervention. Additionally, the changes of the inner vein wall should be observed because the three probiotics consumed glucose in vitro and reduced the glycemic index in the DN mice.

## 5. Conclusions

In conclusion, the probiotic combination of TYCA06, BLI-02, and VDD088 has shown promising results in ameliorating the symptoms of DN, including elevated blood glucose levels, high blood pressure, and the deterioration of renal function. Through their ability to secrete short-chain fatty acids (SCFAs), exhibit antioxidation and anti-inflammatory properties, and consume glucose, these probiotics effectively address the underlying issues associated with DN. Furthermore, as our understanding of the physiological mechanisms of the gut–kidney axis deepens in the future, this probiotic combination holds potential as a novel therapeutic strategy. The findings of this study open up possibilities for an alternative and effective approach to treating DN.

## Data Availability

The datasets used and/or analyzed during the current study are available from the corresponding author on reasonable request.
